# Integrated Valorization of Rice Husk by Pressurized Liquid Extraction: Phenolic-Rich Extract Recovery, Reduced 5-HMF Formation, and Preservation of a Cellulosic Co-Product

**DOI:** 10.3390/antiox15070902

**Published:** 2026-07-21

**Authors:** Milagros Sofia Bustamante-Bernedo, Francisco Paraguay-Delgado, Enrique D. Gomez, Nils Leander Huamán-Castilla

**Affiliations:** 1 Faculty of Sciences, National University of Engineering, Av. Túpac Amaru 210, Lima 15333, Peru; milagros.bustamante.b@uni.pe; 2 Laboratorio de Tecnologías Sustentables para la Extracción de Compuestos de Alto Valor, Instituto de Investigación para el Desarrollo del Perú (IINDEP), Universidad Nacional de Moquegua, Moquegua 18001, Peru; 3 Centro de Investigación en Materiales Avanzados SC (CIMAV), Av. Miguel de Cervantes 120, Chihuahua C.P. 31136, Chihuahua, Mexico; francisco.paraguay@cimav.edu.mx; 4 Department of Chemical Engineering, The Pennsylvania State University, University Park, PA 16802, USA; edg12@psu.edu; 5 Escuela Profesional de Ingeniería Agroindustrial, Universidad Nacional de Moquegua, Moquegua 18001, Peru

**Keywords:** pressurized liquid extraction, rice husk valorization, antioxidant compounds, cellulosic residue, 5-HMF, reducing sugars

## Abstract

Rice husk, a by-product of rice processing, is a natural source of phenolic compounds and cellulose. Although pressurized liquid extraction (PLE) is an efficient technique for recovering antioxidant compounds, its application to rice husk using water–ethanol mixtures and its effect on the chemical and structural properties of the resulting post-extraction lignocellulosic residues remain unexplored. Thus, this study evaluated water–ethanol mixtures (0–60%) at 90–150 °C under PLE conditions. The process was assessed in terms of recovery of antioxidant compounds, co-extraction of reducing sugars, and formation of 5-hydroxymethylfurfural (5-HMF), along with structural and chemical characterization of the post-extraction residue. The highest total phenolic content (3.23 mg GAE/g dry weight) and antioxidant capacity (88.05 μmol TE/g dry weight) were obtained with 60% ethanol at 150 °C, significantly outperforming water-only extraction. The concentrations of gallic acid, caffeic acid, and vanillin in the extracts increased under these conditions. Ethanol played a dual role, acting as an antisolvent that reduced sugar co-extraction while simultaneously suppressing 5-HMF formation. SEM images of the post-extraction residue revealed cell wall disruption at higher temperatures and ethanol concentrations, consistent with the release of phenolic compounds. XRD and FTIR confirmed that the native cellulose I structure and characteristic functional groups were preserved across all extraction conditions. This approach supports the integrated valorization of rice husk by simultaneously producing phenolic-rich extracts and a structurally preserved cellulosic co-product.

## 1. Introduction

Rice (*Oryza sativa* L.) is globally recognized as the most important staple food crop, providing a significant portion of daily calories and essential nutrients [1]. In 2024, global rice production reached approximately 820 million metric tons, with Asia and the Americas accounting for about 89% and 5% of the total, respectively [2]. During the 2024/2025 period, the United States produced about 7.1 million metric tons, while Peru produced approximately 2.5 million metric tons [3]. However, rice processing entails several challenges, including the generation of residues such as rice husk [4].

Rice husk represents approximately 20% of the total grain weight and is generated during milling, which is considered a low-value residue and poses an environmental management problem [5]. Nevertheless, rice husk is a natural source of antioxidant compounds and polymers such as cellulose, whose bioactive and technological properties have attracted interest in the food and pharmaceutical industries [6,7].

Among these antioxidant compounds, polyphenols are the main constituents of rice husk, comprising a diverse group of specific phenolic acids, such as ferulic, vanillic, p-coumaric, gallic, and caffeic acids [8], as well as phenolic aldehydes, such as vanillin [9]. These compounds exhibited high in vitro antioxidant capacity due to their ability to donate hydrogen atoms to neutralize biological free radicals [10,11]. Thus, the recovery of these polyphenols could not only reduce agro-industrial waste but also enable the development of value-added products.

Emerging methods for polyphenol recovery, such as ultrasound-assisted extraction, microwave-assisted extraction, supercritical fluid extraction, and pressurized liquid extraction (PLE), offer several advantages over conventional approaches [12]. In particular, PLE operates under subcritical conditions (10 MPa) at high temperatures (75–200 °C), which maintain the solvent in a liquid state, reduce processing time, and improve polyphenol extraction efficiency [13,14]. However, polyphenol extraction at temperatures above 120 °C often results in the co-extraction of undesirable compounds such as reducing sugars and the formation of 5-hydroxymethylfurfural (5-HMF), which may compromise the stability, safety, and functional performance of the extracts [15]. In addition, 5-HMF has been associated with cytotoxic and carcinogenic effects at high concentrations [16].

Although supercritical CO_2_ extraction using ethanol–water mixtures has been applied to recover polyphenols from rice husk [6], this approach requires high operating pressures and specialized equipment, thereby increasing process complexity and operational costs [17]. In contrast, PLE represents a more accessible alternative due to its lower pressure requirements, shorter extraction times, and higher extraction efficiency. Additionally, unlike SFE, PLE produces a liquid extract that requires subsequent concentration and, depending on the intended application, an additional drying step [18,19]. However, studies on rice husk under subcritical conditions have been limited to water as the solvent, despite ethanol being reported as necessary for selective extraction and enhanced polyphenol solubility [20]. Moreover, the simultaneous effect of this solvent on the co-extraction of reducing sugars and the formation of 5-HMF during PLE of rice husk remains unexplored.

After the PLE, the remaining rice husk matrix contains up to 20% cellulose, a linear polymer of glucose units linked by β-(1,4) bonds [21], with broad industrial applicability in bioenergy, polymers, and nano-cellulosic materials, making it attractive for the production of biodegradable films [22]. Thus, the structural characterization of this residual fraction after PLE can support a more integral valorization of rice husk and align with circular bioeconomy principles.

Herein, the objective was to evaluate a dual-stage sustainable strategy for the valorization of rice husk. First, we analyzed the effect of water–ethanol mixtures (0–60%) at high temperatures (90–150 °C) on the recovery of polyphenols and unwanted compounds (reducing sugars and 5-hydroxymethylfurfural) from rice husk during PLE. Subsequently, the residual rice husk biomass was characterized to assess how the extraction parameters affected the lignocellulosic matrix. The structural and chemical modifications were analyzed using scanning electron microscopy (SEM), X-ray diffraction (XRD), and Fourier-transform infrared spectroscopy (FTIR).

## 2. Materials and Methods

### 2.1. Materials

Rice husk samples (~15 kg) were kindly provided by Linea Verde Soluciones Agropecuarias S.R.L., Valle de Camaná (Arequipa, Peru).

Reagents such as Folin–Ciocalteu (2N), sodium carbonate (95%), DPPH (2,2-diphenyl-1-picrylhydrazyl) (≥90%), AAPH (2,2′-azobis (2-methyl-propanimidamide) dihydrochloride) (≥97%), fluorescein (≥95%), Trolox (≥98%), monopotassium phosphate (≥99%), and dipotassium phosphate (≥99%) were used. Solvents such as ethanol (≥99.9%), methanol (≥99.9%), acetonitrile (≥99.9%), formic acid (≥98%), and specific polyphenols (standards) such as gallic acid (99%), caffeic acid (99%), and vanillin (99%) were purchased from Sigma Aldrich Co., Ltd. (St. Louis, MO, USA).

### 2.2. Sample Preparation

Rice husks were washed with distilled water and dried in a convection oven at 40 °C. The dried material was ground into a powder using a grinder (GR-PV40B, Grondoy, Lima, Peru). The resulting husk powder was sieved through 40- and 60-mesh screens to obtain particles between 250 and 425 µm. After milling, the biomass was stored under refrigeration (10 °C) until further analysis.

### 2.3. Pressurized Liquid Extraction (PLE)

The methodology proposed by Huaman-Castilla et al. [23] was utilized with some modifications. In brief, 4 g of the sample was mixed with 50 g of quartz sand. The mixture was placed into a 100 mL extraction cell with a 30 g base of quartz sand. The extraction process was performed using a water–ethanol mixture (0–60%) at high temperatures (90–150 °C) in a pressurized liquid extraction (PLE) device (ASE 150, Dionex, Thermo Fisher, San Jose, CA, USA). Fixed extraction parameters included 10 MPa pressure, one extraction cycle, 20% rinse volume, a 250 s nitrogen purge time, and a static extraction time of 5 min. These settings yielded a solid-to-liquid ratio of 1:22.5. Additionally, a conventional solid–liquid extraction was performed as a reference extraction method, using 60% methanol at 26 °C for 1 h and 400 rpm under atmospheric conditions. The samples were then collected and stored in amber vials at −20 °C for later chemical analysis.

### 2.4. In Vitro Antioxidants Assays

#### 2.4.1. Total Polyphenol Content (TPC)

TPC was determined using the Folin–Ciocalteu method, as described by Felix et al. [24]. For the assay, diluted extract (20 μL) was added to distilled water (150 μL), followed by the addition of Folin–Ciocalteu reagent (10 μL, 1 N). After 3 min, sodium carbonate solution (20 μL, 10%) was incorporated. The reaction was maintained for 1 h, and absorbance was measured at 765 nm using a Microplate Reader (Synergy/HTX, Biotek Instruments Inc., Winooski, VT, USA). A standard curve of gallic acid (0.02 to 0.08 mg/mL) was prepared simultaneously. TPC was expressed as milligrams of gallic acid equivalent per gram of dry weight (mg GAE/g dw).

#### 2.4.2. Antioxidant Capacity by DPPH (2,2-Diphenyl-1-Picrylhydrazyl) Analysis

The DPPH radical scavenging activity was evaluated according to the method described by Brand-Williams et al. [25]. For the assay, 80 µL of various extract dilutions were mixed with 240 µL of a 50.7 µM DPPH solution and incubated in the dark at room temperature for 30 min. Absorbance was then measured at 517 nm using a microplate reader. Methanol mixed with DPPH was used as the negative control. Antioxidant activity was expressed as IC_50_ values (mg/mL), defined as the concentration of the extract required to reduce the DPPH radical by 50%.

#### 2.4.3. Antioxidant Capacity by Oxygen Radical Absorbance Capacity (ORAC) Analysis

ORAC analyses of the obtained extracts were conducted in a microplate reader (Synergy/HTX, BioTek Instruments Inc., Winooski, VT, USA) according to the methodology proposed by Chirinos et al. [26]. A phosphate-buffered saline solution (PBS, 75 mM, pH 7.4) was used to prepare the AAPH solution (153 mM), fluorescein (55 nM), and trolox calibration standards, and to dilute the extracts. AAPH was used as a peroxyl radical generator to induce the oxidative degradation of fluorescein. Trolox standards were prepared at concentrations of 8, 16, 24, 32, and 40 µM. Fluorescence was measured at excitation and emission wavelengths of 485 and 520 nm. The results were calculated using the area under the curve and expressed as µmol TE (Trolox Equivalent) per g of dry rice husk (µmol TE/g dw).

### 2.5. Quantification of Target Polyphenols

The polyphenol profile was determined using the method suggested by Huaman-Castilla et al. [27], with some modifications. The polyphenol analysis was performed on a Vanquish UHPLC system (Thermo Scientific, Germering, Germany) connected to an Orbitrap Exploris 120 mass spectrometer (Thermo Scientific, Bremen, Germany), equipped with a heated electrospray ionization (HESI) source. Prior to injection, the samples were filtered through a 0.22 µm membrane filter and transferred into HPLC vials. Chromatographic separation was carried out on a C18 column (InfinityLab Poroshell 120, 2.1 mm internal diameter × 150 mm length, 2.7 µm particle size; Agilent Technologies, USA) maintained at 30 °C, with a flow rate of 0.3 mL/min and an injection volume of 2 µL. The mobile phase consisted of water containing 0.1% formic acid (A) and acetonitrile with 0.1% formic acid (B). The gradient was: 0 min, 95% A/5% B; 6 min, 88% A/12% B; 8 min, 74% A/26% B; 10 min, 60% A/40% B; 20 min, 95% A/5% B; and 25 min, 95% A/5% B. The total run time was 25 min. The quantification of specific polyphenols was performed using calibration curves within the range of 0.1–10 µg/mL. The MS analysis was conducted in negative ionization mode, and data acquisition was performed using Full Scan mode at a high resolution (60,000). The quantification of polyphenols was performed using calibration curves prepared from gallic acid, caffeic acid, and vanillin standards. Further details are provided in the Supplementary Materials.

### 2.6. 5-Hydroxymethylfurfural (5-HMF)

The analysis of 5-HMF levels in the extract was carried out according to the method reported by Wang et al. [28]. Filtered samples were analyzed using a Vanquish UHPLC system (Thermo Scientific, Germering, Germany) connected to an Orbitrap Exploris 120 mass spectrometer (Thermo Scientific, Bremen, Germany), equipped with a heated electrospray ionization (HESI) source. The chromatographic separation of 5-HMF was conducted on an Agilent HC-C18 column (4.6 mm internal diameter × 250 mm length, Agilent Technologies, Netherlands) at a flow rate of 0.3 mL/min. The mobile phase was methanol (A) and distilled water containing 0.2% (*v*/*v*) formic acid (B). The gradient was: 0 min, 5% A/95% B; 3 min, 20% A/80% B; 8 min, 5% A/95% B; 12 min, 5% A/95% B. The total run time was 12 min. The injection volume was 5 μL, and the column temperature was maintained at 30 °C. Results were expressed as µg of 5-HMF per 100 g of dry rice husk.

### 2.7. Sugar Content

The methodology proposed by Debebe et al. [29] was used to determine the contents of sucrose, glucose, and fructose, with some modifications. Soluble sugars were analyzed by ultra-high-performance liquid chromatography (UHPLC) (Agilent 1290 Infinity II, Santa Clara, CA, USA), equipped with a refractive index detector (RID G7162A). The rice husk extract was filtered through a 0.22 µm membrane filter and directly transferred to HPLC vials without additional treatment. Chromatographic separation was performed on a Hi-Plex H column (300 × 6.5 mm, particle size 8 µm, Agilent Technologies, United Kingdom) maintained at 50 °C, using ultrapure water type I as the mobile phase at a flow rate of 0.6 mL/min under isocratic conditions. The injection volume was 10 μL, and the total run time was 15 min. After preparation, the mobile phase was filtered under vacuum using cellulose esters with a 47 mm diameter and a 0.45 μm porosity and degassed in an ultrasonic bath. Identification of glucose, fructose, and sucrose was achieved by comparing retention times with standards, whereas quantification was performed using external calibration curves prepared with glucose, fructose, and sucrose standards over the range of 0.2–1 mg/mL (R^2^ = 0.9998). The results were expressed as mg of glucose, fructose, or sucrose per g of dry rice husk.

### 2.8. X-Ray Diffraction

The X-ray diffraction (XRD) pattern of the extract-free husk samples was measured using a Rigaku MiniFlex 600 X-ray diffractometer (Rigaku Corporation, Tokyo, Japan) operated at 40 kV and 15 mA with Cu Kα radiation (α = 1.5418 Å). Diffractograms were captured between 5 and 60° at a scan speed of 2°/min with a step size of 0.02.

### 2.9. Scanning Electron Microscopy (SEM)

The morphological properties of the powder were investigated by scanning electron microscopy (SEM) (SU3500; HITACHI, Tokyo, Japan). Samples were affixed to carbon conductive tabs without additional coating. Imaging was conducted under two detection modes. Using the ultra-variable-pressure detector (UVD, 60 Pa), micrographs were obtained at 50×, 150×, and 500× using an accelerating voltage of 15.0 kV and in secondary electron (SE) mode. Images were captured at 1.00 k× and 2.50 k× with an accelerating voltage of 5.00 kV.

### 2.10. Attenuated Total Reflectance—Fourier Transform Infrared Spectroscopy (ATR-FTIR)

The identification of functional groups in untreated and pretreated rice husk (RH) was conducted using a FTIR spectrophotometer (Vertex 70v, Bruker, Billerica, MA, USA) equipped with a liquid-nitrogen-cooled mercury–cadmium–telluride (MCT) detector. Measurements were performed in attenuated total reflectance (ATR) mode using a Harrick Diamax diamond single-bounce ATR accessory. A total of 400 scans were averaged at a resolution of 6 cm^−1^, and reflectance was calculated by referencing to a clean bare diamond crystal.

### 2.11. Statistical Analysis

Data are expressed as mean ± standard deviation (SD). A full factorial experimental design (3 × 3) was used to evaluate the main and interaction effects of the studied factors on the response variables. The significance of the main factors and their interactions was assessed by analysis of variance (ANOVA), and mean comparisons were performed using Tukey’s multiple range test at a significance level of *p* < 0.05. Statistical analyses of the data were performed using OriginPro 2026 (OriginLab, Northampton, MA, USA).

## 3. Results and Discussion

### 3.1. Recovery of Antioxidant Compounds from Rice Husk

#### 3.1.1. Total Phenolic Content (TPC)

**Figure 1:**
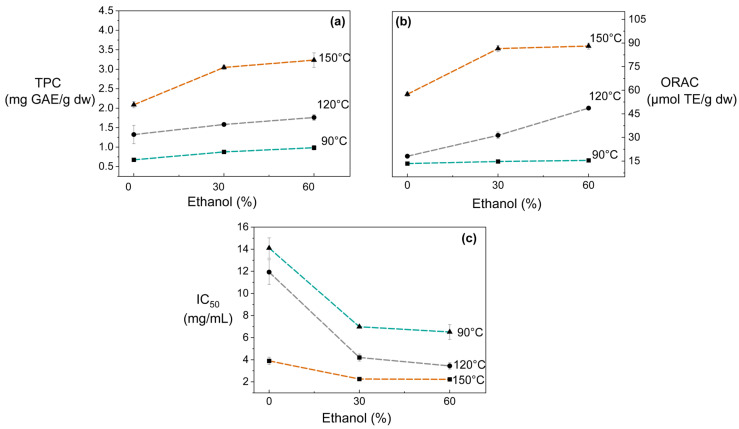
Effect of extraction temperature and ethanol concentration on (**a**) total phenolic content (TPC) and antioxidant activity, as determined by (**b**) ORAC and (**c**) DPPH assays.

Regardless of the solvent composition, high temperatures enhance the recovery of total phenolic content (TPC) from rice husk (*p* < 0.05) (Figure 1). For example, increasing the extraction temperature from 90 to 150 °C enhanced TPC by 1.4, 2.2, and 2.3 mg GAE/g for water, 30% ethanol, and 60% ethanol, respectively (Figure 1). These results are consistent with previous work on coffee bean husk. Costa et al. [30] demonstrated that increasing the temperature from 40 to 80 °C with 75% ethanol under PLE enhanced TPC extractability from 75.1 to 86.6 mg GAE/g of dry extract. At 60 °C, increasing the ethanol concentration from 50% to 100% increased polyphenol recovery from 79.4 to 98.2 mg GAE/g of dry extract. Similarly, Teixeira et al. [31] reported that, in black bean husk extracts, increasing the temperature from 30 to 70 °C and the ethanol concentration from 30 to 50% increased the TPC from 16.7 to 18.4 mg GAE/g dw. Dobroslavić et al. [32] reported a 44.6% increase in TPC when the temperature was increased from 90 °C to 150 °C with 50% ethanol from *Laurus nobilis* leaves. Although the Folin–Ciocalteu assay is widely accepted as a standard approach for phenolic quantification, it is not entirely selective for phenolic compounds. Other reducing constituents present in the extract (e.g., ascorbic acid, reducing sugars, and amino acids) may also react with the reagent, contributing to the measured response and potentially leading to an overestimation of TPC values. In this sense, the TPC results should be interpreted as an estimate of the overall reducing capacity of the extract rather than an absolute measure of phenolic content [33].

Water–ethanol mixtures have proven to be highly effective solvents for maximizing extraction yields from plant matrices, particularly under subcritical conditions [34]. An increase in temperature reduces the solvent viscosity and surface tension, thereby enhancing diffusivity and facilitating greater penetration into the plant matrix [35]. The polarity of the solvent should be similar to that of the target compounds to maximize solubility and facilitate mass transfer [36].

Polyphenols have both hydrophilic and hydrophobic structural domains, which facilitate hydrophobic interactions and hydrogen bonding with the hydroxyl and methyl groups of ethanol [37]. Nevertheless, under subcritical conditions, ethanol’s ability to form hydrogen bonds decreases, as reflected in its solvatochromic parameters. The hydrogen-bond donating acidity (α) decreases from 0.83 to 0.52, and the hydrogen-bond accepting basicity (β) decreases from 0.75 to 0.16, alongside a reduction in the polarity/polarizability (π*) from 0.51 to −0.03 [38]. These changes confirm a decline in ethanol’s ability to form hydrogen bonds. As a result, nonpolar interactions (London dispersion forces) increase, creating an environment that favors the extraction of less polar phenolic compounds under subcritical conditions [39].

#### 3.1.2. Antioxidant Capacity

The antioxidant capacity of the rice husk extracts was evaluated using the ORAC and DPPH assays (Figure 1). The DPPH assay measures the ability of polyphenols to neutralize a synthetic radical [40], while the ORAC assay quantifies the inhibition of peroxyl radicals, which are biologically relevant in food and physiological systems [41]. Additionally, there is a positive correlation between total phenolic content and ORAC values, whereas DPPH results are expressed as IC_50_ values, which are inversely proportional to phenolic content [42].

The extract obtained with 60% ethanol at 150 °C presents the highest antioxidant activity (88.05 μmol TE/g dw) measured by ORAC, similar to 30% ethanol (86.52 μmol TE/g dw), but significantly higher than pure water (57.45 μmol TE/g dw) (*p* < 0.05) (Figure 1b). Similarly, under subcritical conditions, Alvarez et al. [43] demonstrated that the use of pure ethanol combined with an increase in temperature from 25 to 180 °C enhanced the antioxidant capacity from 18.15 to 77.18 millimolar Trolox equivalents per milligram (mMET/mg) from oak leaves.

On the other hand, DPPH assays show that using 60% ethanol at 150 °C yields the lowest IC_50_ (2.23 mg/mL), indicating the highest free radical-scavenging capacity, whereas water at 90 °C shows the weakest antioxidant activity (IC_50_ = 14.11 mg/mL) (*p* < 0.05) (Figure 1c). These results are consistent with those of Ballesteros-Vivas et al. [44], who showed that increasing the temperature from 50 to 150 °C and the ethanol concentration from 0 to 100% in PLE of mango seed kernels reduces the IC_50_ value from 42.51 µg/mL to 17.59 µg/mL.

#### 3.1.3. Comparison Between Rice Husk Extracted by PLE and Conventional Solid–Liquid Extraction

PLE exhibits higher efficiency in recovering phenolic compounds than the conventional extraction (CE) method used as a reference (*p* < 0.05). Under CE conditions (60% methanol at 26 °C, 1 h), a TPC of 0.47 mg GAE/g was obtained, whereas PLE with 60% ethanol at 150 °C yielded a higher TPC of 3.23 mg GAE/g. Olszowy-Tomczyk and Wianowska et al. [45] evaluated walnut husks by maceration and PLE using methanol, finding that maceration recovered 41.6% less polyphenols than PLE.

Antioxidant activity followed the same trend. Extracts from the CE process yielded an oxygen radical absorbance capacity (ORAC) value of 32.89 µmol TE/g dry sample and a DPPH IC_50_ of 14.93 mg/mL, both of which were significantly lower than the optimal PLE conditions (150 °C, 60% ethanol). The combination of higher IC_50_ values and lower ORAC values indicates reduced radical-scavenging efficiency and confirms the enhanced antioxidant activity of PLE extracts.

These results confirm that PLE enhances the accessibility of bioactive compounds within the plant matrix by improving solvent diffusivity and mass transfer kinetics, thereby facilitating solvent penetration and accelerating mass transfer into the lignocellulosic structure. Consequently, PLE enables higher recoveries of bioactive compounds in shorter extraction times and with reduced solvent consumption than conventional methods [46,47].

#### 3.1.4. Polyphenolic Profile

**Figure 2:**
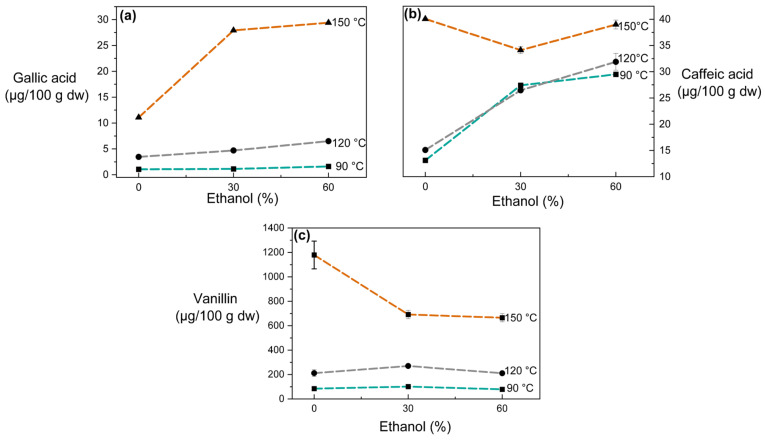
Recovery of phenolic compounds using PLE of the extracts obtained from rice husk at different temperatures and ethanol concentrations: (**a**) gallic acid (µg/100 g dw), (**b**) caffeic acid (µg/100 g dw), and (**c**) vanillin (µg/100 g dw).

Three phenolic compounds, caffeic acid, gallic acid, and vanillin, were identified and quantified in the rice husk extracts obtained by PLE. Overall, increasing the extraction temperature from 90 to 150 °C promoted the recovery of all identified compounds (Figure 2); however, the effect of ethanol concentration was compound-dependent, reflecting the different molecular structures and polarities of the phenolics.

For caffeic and gallic acids, the extraction efficiency under PLE conditions is controlled by temperature (*p* < 0.05). During PLE, when temperature increases from 90 to 150 °C using pure water, 30% ethanol, and 60% ethanol, the recovery of caffeic acid increases by 3.1, 1.2, and 1.3 times, respectively (Figure 2b). Similarly, gallic acid recovery increases by 10, 25, and 18 times under the same conditions (Figure 2a). Huaman-Castilla et al. [27] reported increased recovery of phenolic acids, such as gallic and caffeic acids, as temperature increased from 90 to 150 °C, reaching maximum values at 50% ethanol and 150 °C.

At 150 °C, vanillin increases by 14, 7, and 8 times for 0%, 30%, and 60% ethanol, respectively, compared with 90 °C (*p* < 0.05) (Figure 2c). The effect of ethanol concentration was compound-dependent; in the case of vanillin, pure water yields the highest recovery at 150 °C, whereas increasing ethanol content leads to a pronounced decrease in vanillin extraction. Buranov and Mazza [48] demonstrated that subcritical water at 220 °C increased vanillin recovery from flax shives relative to pressurized aqueous ethanol, yielding 102 mg/100 g compared to 91 mg/100 g, respectively. These data are consistent with previous reports of phenolic acid composition in products extracted from rice husk [6,7,9].

This trend is consistent with the findings of Setyaningsih et al. [49], which evaluated the influence of key PLE operational variables, including solvent composition and extraction temperature, on the yield of phenolic compounds from rice grains. Among these variables, temperature was the most influential factor, resulting in a pronounced increase in phenolic recovery with increasing temperature, with maximum yields observed at approximately 190 °C, whereas solvent composition exerted a comparatively smaller effect.

#### 3.1.5. Reducing Sugars and 5-HMF Content

**Figure 3:**
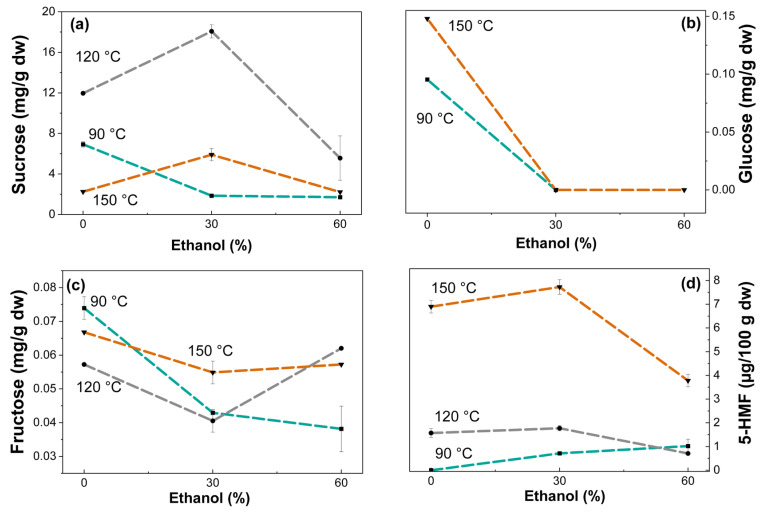
Sugar and 5-HMF yield after PLE. The (**a**) sucrose, (**b**) glucose, and (**c**) fructose content is expressed as milligrams per gram of dry weight (mg/g dw), and (**d**) 5-HMF (µg/100 g dw).

During the PLE, both solvent composition and temperature influence the recovery of reducing sugars (sucrose, glucose, and fructose). For example, when temperature increased from 90 to 120 °C, sucrose recovery increased by 5, 16, and 4 mg/g for pure water, 30% ethanol, and 60% ethanol (*p* < 0.05), respectively (Figure 3a). Conversely, high temperatures (150 °C) decreased sucrose extraction in all three solvent systems, likely due to thermal hydrolysis of the glycosidic bond, yielding glucose and fructose monomers (Figure 3). Under aqueous extraction conditions, glucose was detected at relatively low concentrations; however, increasing the extraction temperature from 90 to 150 °C increased glucose content from 0.095 to 0.148 mg/g. In contrast, glucose was not detected by the analytical method in extracts obtained using 30% or 60% ethanol within the studied temperature range (Figure 3b). Fructose concentrations remained low, ranging from approximately 0.04 to 0.07 mg/g, across all solvents and temperatures evaluated (Figure 3c).

Huamán-Castilla et al. [50] demonstrated that increasing ethanol concentration during PLE reduces the recovery of reducing sugars. Specifically, using 30% ethanol at 70 °C decreased glucose and fructose content by 40% and 34%, respectively, compared with aqueous extraction. Moreover, Abaide et al. [51] demonstrated that pressurized hot water extraction (25 MPa) combined with an increase from 180 to 220 °C can hydrolyze cellulose, increasing glucose concentration from 0.3 to 1.5 g/L from rice husk extracts. Similarly, Lachos-Perez et al. [52] reported that during subcritical water treatment of sugarcane straw, glucose yield increased from 1.5 to 2.1 g/100 g as temperature rose from 190 to 200 °C, but decreased at higher temperatures (225–260 °C). The presence of glucose in the aqueous extracts can be attributed to the preferential hydrolysis of the amorphous regions of cellulose, where glycosidic bonds are more readily accessible. This hydrolysis is promoted by subcritical water through cleavage of glycosidic bonds in cellulose, with higher temperatures increasing the hydronium ion concentration and thereby accelerating the reaction rate [52]. In addition to temperature effects, the addition of ethanol reduces solvent polarity and acts as an antisolvent, weakening hydrogen-bonding interactions and markedly lowering glucose solubility [53].

5-Hydroxymethylfurfural (5-HMF) concentrations increased with temperature from 90 to 150 °C across all solvent systems evaluated (*p* < 0.05), whereas increasing the ethanol concentration from 0% to 60% at 150 °C led to a 45.2% decrease in 5-HMF (Figure 3d). This compound is formed by sugar dehydration during thermochemical hydrolysis of rice husk, and its accumulation increases at higher temperatures, resulting in elevated concentrations in the hydrolysate [54]. 5-HMF is considered an undesirable byproduct, as it compromises the quality of rice husk hydrolysates and limits their potential use in food applications. The 5-HMF content was expressed on a dry biomass basis (µg/100 g dry rice husk) to indicate the amount recovered per unit of initial biomass and provide a mass-normalized value for comparison with other studies, while µg/L represents the concentration directly quantified in the liquid extract. Although no specific 5-HMF limit has been established for lignocellulosic extracts, the concentration measured in the rice husk extract (1.471–16.658 µg/L) was markedly lower than the reference limit for regulated food matrices such as fruit juice (10 mg/L) [55], indicating a low food-safety risk. Increasing the ethanol content to 60% in PLE reduced 5-HMF formation, which may be explained by a proposed mechanism in which lower solvent polarity limits sugar dehydration reactions without compromising the recovery of phenolic compounds. A similar trend has been reported in other lignocellulosic matrices, such as coffee grounds, where higher ethanol fractions during extraction decreased 5-HMF levels by up to 50% [56].

### 3.2. Characterization of the Residual Biomass After the PLE Process

After evaluating the effect of the PLE conditions on the yields of antioxidant compounds, the lignocellulosic matrix in the resulting PLE residue was analyzed for its morphology and microstructure.

#### 3.2.1. Scanning Electron Microscopy (SEM) of Rice Husk and Residual Biomass

**Figure 4:**
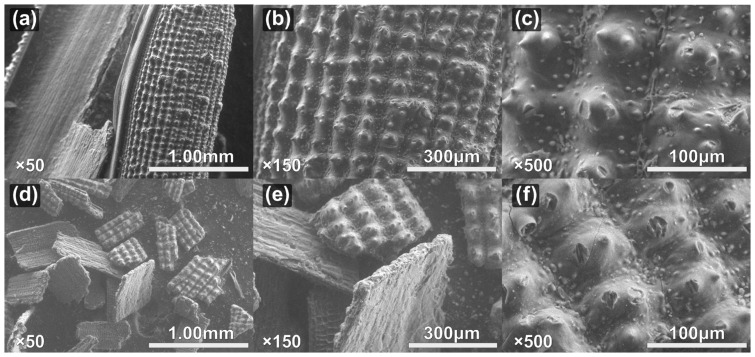
SEM of the outer surface of rice husk. Figures (**a**–**c**) represent images of the untreated sample at magnifications of ×50, ×150, and ×500, respectively. Figures (**d**–**f**) represent images of the ground husk fractions retained between 40 and 60 mesh sieves at the same magnifications, respectively. Micrographs were acquired at 15 kV in low-vacuum mode (UVD, 60 Pa).

Figure 4a–f shows scanning electron micrographs of the outer surface of rice husk (RH) at different magnifications, before (a–c) and after (d–f) mechanical grinding. At low magnification (Figure 4a, ×50), the rice husk surface appears as elongated, continuous epidermal structures organized into longitudinal ridges and furrows that define the outer epidermis. At medium magnification (Figure 4b, ×150), a well-defined, repetitive ridged pattern becomes evident, formed by alternating ridges and furrows. At higher magnification (Figure 4c, ×500), the surface reveals a dense distribution of micro-bumps and conical protrusions distributed along the ridged epidermis, resulting in a corrugated, textured outer surface typical of the silica-rich epidermis of rice husk.

After mechanical grinding (Figure 4d–f), the RH fragments preserve the main morphological features of the outer epidermis. At ×50 magnification (Figure 4d), the continuous ridged structure appears fragmented into irregular particles. At ×150 magnification (Figure 4e), traces of the original ridged pattern and conical protrusions remain discernible. At ×500 magnification (Figure 4f), the ridge alignment and protrusions persist on the fragments. These results are consistent with the hierarchical structure of rice husk and the localization of silica in the outer epidermis described in previous works [57,58].

These morphological characteristics are consistent with previous reports on raw rice husk, which describe a surface composition predominantly of carbon, oxygen, and silica, as evidenced by SEM–EDX analysis [59,60]. In the present study, energy-dispersive X-ray spectroscopy of the outer surface revealed elemental contents of 29.1–37.3% carbon, 39.6–44.2% oxygen, 22.6–27.7% silicon, 0.2–0.7% aluminum, and 0.1–0.4% potassium by weight, confirming the silica-enriched nature of the outer epidermis and the minor presence of other inorganic elements.

**Figure 5:**
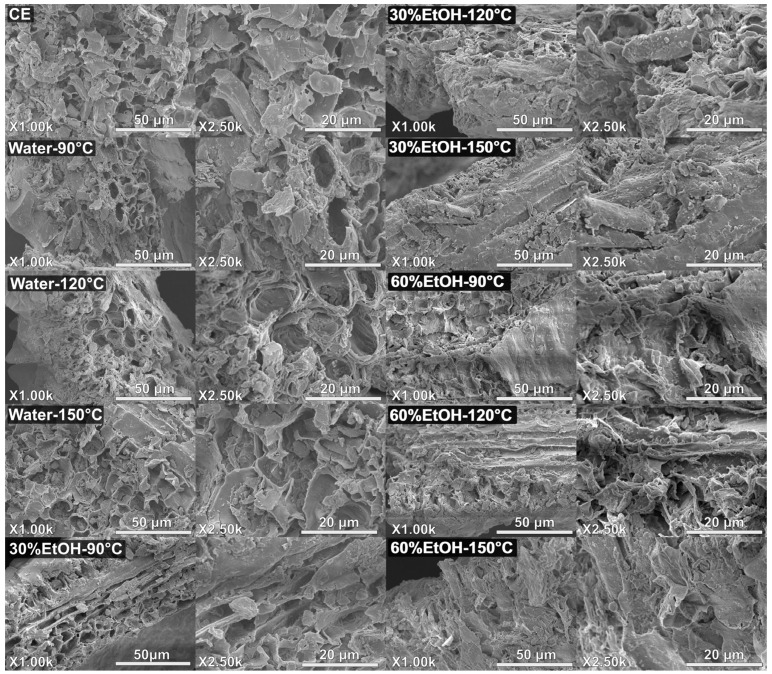
Scanning electron microscopy (SEM) images of biomass under different extraction conditions from rice husk. CE: conventional extraction; PLE with water (90–120–150 °C); PLE with 30% ethanol (90–120–150 °C); PLE with 60% ethanol (90–120–150 °C). Micrographs were acquired at 5 kV using a secondary electron (SE) detector.

After CE, the cell walls remained continuous and intact, with no visible signs of fragmentation or detachment (Figure 5). This suggests limited solvent–matrix interactions under mild extraction conditions. In contrast, PLE samples showed progressive cell wall disruption as both temperature and ethanol concentration increased. Water-treated samples retained continuous cell walls across all conditions, with only minor cavities opening at 150 °C. At 30% ethanol, the cell walls were partially altered at all temperatures. At 60% ethanol, deformation became more pronounced, and the original cell wall shape was lost, with the most severe disruption of the cell wall structure observed at 60% ethanol combined with 150 °C (Figure 5). These morphological changes are associated with greater surface exposure, enhanced solvent diffusivity, and improved matrix permeability, which facilitates the release of phenolic compounds primarily bound to the cell wall [61].

Comparable structural alterations have been reported in other matrices subjected to pressurized extraction. Toda et al. [62] observed surface openings in spent coffee grounds after PLE, attributed to solvent pathways formed within the matrix and to cell wall damage induced by the combined effect of pressure and temperature. Similarly, Liang et al. [63] recovered polyphenols from *Phyllanthus emblica* pomace using subcritical water (200 °C, 5 MPa, 25 min) and found that the cellular framework of the post-extraction residue collapsed into a porous, sponge-like matrix, in parallel with an increase in polyphenol yield compared with conventional solvent extraction. Zakaria et al. [64] reported complete rupture of Chlorella sp. cells, originally rounded and agglomerated, after subcritical water treatment (163 °C, 5 min). In line with these findings, Zhang et al. [65] described greater cell wall distortion in *Carpophyllum flexuosum* after pressurized hot water extraction (160 °C) than after room-temperature aqueous extraction, which was consistent with the higher phlorotannin recovery achieved under pressurized conditions.

Overall, these observations are consistent with a progressive reduction in cell-wall integrity with increasing temperature and solvent polarity, which may enhance solvent penetration and the accessibility of phenolic compounds bound to structural carbohydrates and lignin. This qualitative evidence, together with the corresponding phenolic compounds yield and antioxidant capacity results, supports a proposed extraction mechanism under PLE conditions. Quantitative image analysis (e.g., porosity, particle size) could further validate this mechanism in future studies.

#### 3.2.2. XRD

**Figure 6:**
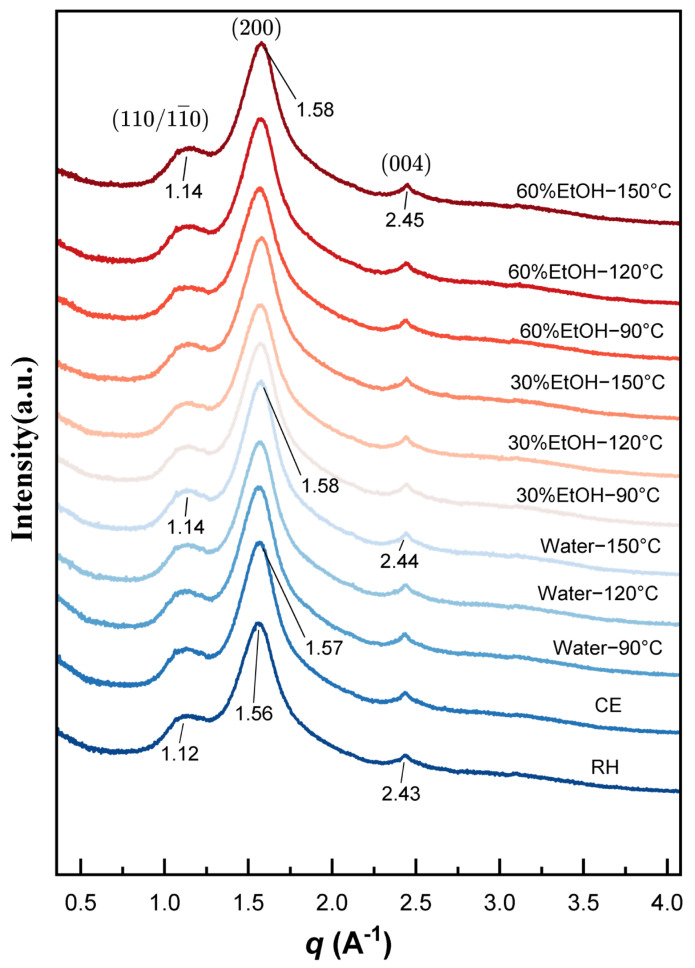
X-ray diffraction patterns of biomass under different extraction conditions from rice husk. CE: conventional extraction; RH: untreated rice husk; PLE with water (90–120–150 °C); PLE with 30% ethanol (90–120–150 °C); PLE with 60% ethanol (90–120–150 °C). Key reflections are denoted with *q* values.

Figure 6 presents the X-ray diffraction (XRD) patterns of rice husk and rice husk under different extraction conditions as X-ray intensity versus scattering vector (*q =* 4*πsin*(*θ*/2)/*λ*, where *θ* is the scattering angle and *λ* is the X-ray wavelength) to normalize for the incident X-ray wavelength. The (110/$1\bar{1}0$), (200), and (004) reflections of RH were observed at 1.12 ${Å}^{-1}$ (~16°), 1.56 ${Å}^{-1}$ (~22°), and 2.43 ${Å}^{-1}$ (~34°) respectively, consistent with the characteristic diffraction peaks of cellulose I [66,67,68]. A reflection from amorphous silica is also expected at ~22° [69]. After PLE at 150 °C, the (200) reflection shifted slightly to higher q values, from 1.56 ${Å}^{-1}$ to 1.58 ${Å}^{-1}$. However, all XRD profiles were similar, indicating that the cellulose crystalline structure was largely preserved across the tested extraction conditions.

These findings are consistent with the study by Yin et al. [70], which showed that the removal of bound polyphenols by alkaline hydrolysis produced only minor changes in the XRD profiles, with both untreated and treated samples retaining the typical cellulose I structure. Similarly, Toda et al. [62] extracted phenolic compounds from spent coffee grounds by ultrasound-assisted extraction (UAE) and observed that the crystalline structure of the solid residue remained unchanged, with the characteristic cellulose peak at ~22° comparable to that of the untreated material. Liu et al. [71] likewise reported that cellulose I preserves its crystalline structure under subcritical water conditions (498 K, 6 MPa) across different reaction times [72].

These results confirm the structural stability of cellulose under PLE, showing that the increase in phenolic compounds recovery at 150 °C is not associated with the formation of additional crystalline phases, as no new peaks were observed in the diffractograms. The absence of significant changes in peak intensity or width can be attributed to the relatively short exposure time (5 min) under high pressure and temperature, which is insufficient to induce notable modifications in the lignocellulosic network. Longer treatment times may alter cellulose crystallinity and, consequently, affect the yield of phenolic compounds recovered from rice husk. Future studies should therefore evaluate the effect of extraction temperature and residence time on cellulose crystallinity under PLE.

#### 3.2.3. ATR-FTIR

**Figure 7:**
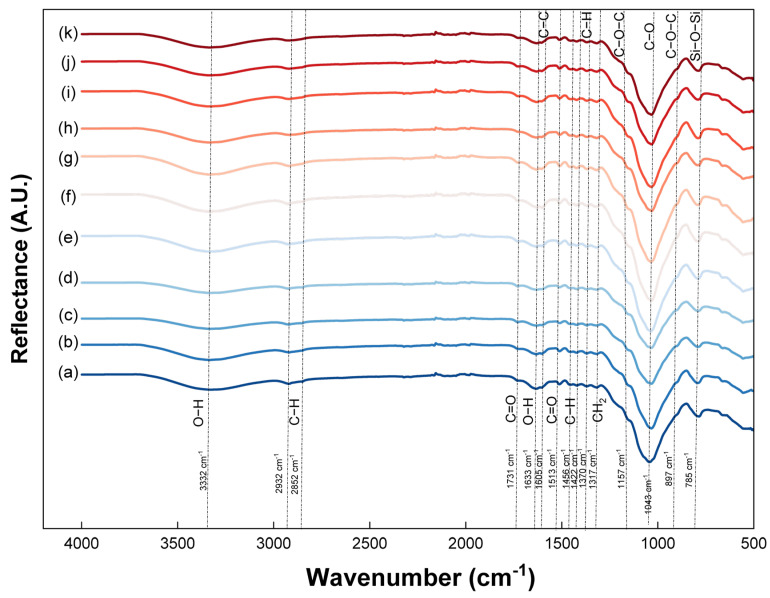
ATR-FTIR spectra of rice husk biomass under different extraction conditions: (a) untreated rice husk (RH); (b) conventional extraction (CE); PLE with water at (c) 90 °C, (d) 120 °C, and (e) 150 °C; PLE with 30% ethanol at (f) 90 °C, (g) 120 °C, and (h) 150 °C; and PLE with 60% ethanol at (i) 90 °C, (j) 120 °C, and (k) 150 °C.

The ATR-FTIR spectra of rice husk (RH) and post-extraction biomass (solid remaining after CE and PLE) are shown in Figure 7. The broad band around 3332 cm^−1^ is attributed to O-H stretching vibrations of hydroxyl groups associated with intramolecular bonds [73] or cellulosic material [74], while the small bands in the range of 2919 to 2932 cm^−1^ and 2852 cm^−1^ are related to the C-H groups of long-chain aliphatic components (alkanes and alkenes structures) of cellulose, lignin, and hemicellulose [75].

The weak absorption band near 1731 cm^−1^ is related to the carbonyl bond (C=O) of hemicellulose [76], and the band around 1633 cm^−1^ corresponds to O-H bending vibrations of adsorbed water within the cellulose structure [77] or to the C=O bond of the uronic acids from hemicellulose [78]. The absorption band at ~1605 cm^−1^ is attributed to the in-plane symmetric C–C stretching vibration of aromatic rings in lignin [79].

The band at 1513 cm^−1^ is associated with aromatic ring and aromatic skeletal vibrations characteristic of lignin [24,80]. Likewise, minor bands at 1456 and 1422 cm^−1^ are associated with aromatic C-H bends in lignin [76], while the peak at 1370 cm^−1^ is related to the C-H deformation in cellulose and hemicellulose [81], and the peak observed near 1317 cm^−1^ is attributed to CH_2_ wagging modes in cellulose [82].

Additionally, a strong band is present at 1043 cm^−1^ is characteristic of cellulose [81]. The band in the range of 1159 to 1162 cm^−1^ is associated with the degree of crystallinity in cellulose I [83] and a shoulder at approximately 897 cm^−1^ is related to β-glycosidic linkages [80] or C-H deformation in cellulose [76]. The band observed at 785 cm^−1^ is assigned to Si–O–Si stretching vibrations, which are characteristic of silica [84].

Sasaki et al. [85] reported that solid residues recovered after subcritical water treatment exhibited FTIR spectra similar to those of the original cellulose, indicating preservation of its macromolecular structure. As previously mentioned, XRD analysis showed that the original crystalline structure was preserved, confirming that the remaining solid fraction corresponds to cellulose. Similarly, Lü and Saka [76] demonstrated that the crystalline structure and the characteristic FTIR peaks of the lignocellulosic biomass remained unchanged within the 170–210 °C range under hot-compressed water treatment. The first structural variation was observed at 230 °C, evidenced by the disappearance of the hemicellulose C=O band (1740 cm^−1^) and the initial disruption of the cellulose crystalline structure.

## 4. Conclusions

This study elucidates the effects of extraction temperature and solvent composition on water–ethanol-assisted PLE for the recovery of selected phenolic compounds from rice husk, while simultaneously evaluating the extraction of reducing sugars and the formation of 5-HMF. The highest total phenolic content (3.23 mg GAE/g dry weight) and antioxidant capacity (88.05 μmol TE/g dry weight) were obtained at 150 °C and 60% ethanol, the highest levels evaluated for both factors. This progressive increase suggests that more severe conditions may further enhance phenolic recovery, although this hypothesis requires validation due to potential degradation of phenolic compounds. In addition, specific phenolic compounds, such as caffeic acid, gallic acid, and vanillin, showed a progressive increase in content with increasing temperature. The use of a high ethanol concentration improved extract quality by suppressing 5-HMF formation and reducing sugar content. SEM images showed progressive fragmentation of the cell wall structure at higher temperatures and in the presence of ethanol in PLE, thereby facilitating the release of phenolic compounds. XRD analysis confirms the preservation of the cellulose I crystalline structure, while FTIR analysis corroborates this by showing the retention of characteristic cellulose functional groups. These findings support an integrated valorization strategy for rice husk, enabling simultaneous recovery of phenolic compound-rich extracts and a cellulose-rich co-product while preserving structural features. These preserved features indicate the potential for further valorization of the residual solid fraction as a cellulosic co-product; however, additional characterization, including thermal stability, compositional analysis, and particle size, would be needed to confirm its suitability for specific applications.

## Data Availability

The original contributions presented in this study are included in the article/Supplementary Materials.

## Supplementary Materials

The following supporting information can be downloaded at: https://www.mdpi.com/article/10.3390/antiox15070902/s1. Table S1: UHPLC-Orbitrap analytical and validation parameters for target phenolic compounds; Table S2: Factorial ANOVA for TPC; Table S3: Factorial ANOVA for ORAC; Table S4: Factorial ANOVA for DPPH.
