# Supplementary material for: Integrated Valorization of Rice Husk by Pressurized Liquid Extraction: Phenolic-Rich Extract Recovery, Reduced 5-HMF Formation, and Preservation of a Cellulosic Co-Product
Source: Antioxidants (Basel). 2026 Jul 21;15(7):902. doi: 10.3390/antiox15070902 (PMC13403786; doi:10.3390/antiox15070902)
Supplement: Supplementary file 1 [file antioxidants-15-00902-s001.zip › antioxidants-4420111-supplementary.pdf]

Supplementary Materials

Table S1. UHPLC-Orbitrap analytical and validation parameters for target phenolic compounds

Target compound	Measured exact mass (m/z)	RT	LOD (µg/mL)	LOQ (µg/mL)	R^2
Gallic acid	169.0153961	2.40	0.001	0.005	0.997
Caffeic acid	179.0361328	9.68	0.002	0.007	0.996
Vanillin	151.0410767	11.08	0.003	0.012	0.997

ANOVA results for the effects of extraction parameters on TPC, ORAC, and DPPH responses

Table S2. Factorial ANOVA for TPC

Source	DF	Adj SS	Adj MS	F-Value	P-Value
Ethanol	2	1.2930	0.64652	52.19	0.000
Temperature	2	11.6173	5.80865	468.93	0.000
Ethanol*Temperature	4	0.5090	0.12724	10.27	0.002
Error	9	0.1115	0.01239		
Total	17	13.5308			

Table S3. Factorial ANOVA for ORAC

Source	DF	Adj SS	Adj MS	F-Value	P-Value
Ethanol	2	1394.7	697.34	350.33	0.000
Temperature	2	12525.8	6262.89	3146.35	0.000
Ethanol*Temperature	4	737.4	184.36	92.62	0.000
Error	9	17.9	1.99		
Total	17	14675.8			

Table S4. Factorial ANOVA for DPPH

Source	DF	Adj SS	Adj MS	F-Value	P-Value
Ethanol	2	130.957	65.4787	207.95	0.000
Temperature	2	124.216	62.1079	197.24	0.000
Ethanol*Temperature	4	33.520	8.3799	26.61	0.000
Error	9	2.834	0.3149		
Total	17	291.527			
